# Sweet Cherries as Anti-Cancer Agents: From Bioactive Compounds to Function

**DOI:** 10.3390/molecules26102941

**Published:** 2021-05-15

**Authors:** Lara R. S. Fonseca, Gonçalo R. Silva, Ângelo Luís, Henrique J. Cardoso, Sara Correia, Cátia V. Vaz, Ana P. Duarte, Sílvia Socorro

**Affiliations:** 1 CICS-UBI—Health Sciences Research Centre, University of Beira Interior, Av. Infante D. Henrique, 6200-501 Covilhã, Portugal; lara.fonseca@ubi.pt (L.R.S.F.); afluis27@gmail.com (Â.L.); henrique10mc@gmail.com (H.J.C.); scorreia@fcsaude.ubi.pt (S.C.); d793@fcsaude.ubi.pt (C.V.V.); 2 School of Biological Sciences, Queen’s University Belfast, Belfast BT9 5DL, UK; grosasdasilva01@qub.ac.uk

**Keywords:** sweet cherries, anthocyanins, anti-cancer agents, oxidative stress, inflammation, proliferation, apoptosis, invasion, metastization, metabolic reprogramming

## Abstract

Sweet cherries (*Prunus avium* L.) are among the most appreciated fruits worldwide because of their organoleptic properties and nutritional value. The accurate phytochemical composition and nutritional value of sweet cherries depends on the climatic region, cultivar, and bioaccessibility and bioavailability of specific compounds. Nevertheless, sweet cherry extracts are highly enriched in several phenolic compounds with relevant bioactivity. Over the years, technological advances in chemical analysis and fields as varied as proteomics, genomics and bioinformatics, have allowed the detailed characterization of the sweet cherry bioactive phytonutrients and their biological function. In this context, the effect of sweet cherries on suppressing important events in the carcinogenic process, such as oxidative stress and inflammation, was widely documented. Interestingly, results from our research group and others have widened the action of sweet cherries to many hallmarks of cancer, namely metabolic reprogramming. The present review discusses the anticarcinogenic potential of sweet cherries by addressing their phytochemical composition, the bioaccessibility and bioavailability of specific bioactive compounds, and the existing knowledge concerning the effects against oxidative stress, chronic inflammation, deregulated cell proliferation and apoptosis, invasion and metastization, and metabolic alterations. Globally, this review highlights the prospective use of sweet cherries as a dietary supplement or in cancer treatment.

## 1. Introduction

Plant-based natural medicines are unquestionably humanity’s oldest and longest-abiding source of health treatments, as well as some of the most versatile [1,2,3]. Despite being faded out in favor of other medicinal processes as technology progressed, the use of natural products in the pharmacological pipeline has remained important and even undergone a resurgence [2]. This includes the application of plant-based medicines in the field of cancer research and treatment [4]. In the last few decades, preventive and generalized chemotherapeutic cancer treatments have been achieved from plant and fruit extracts [5,6]. Moreover, the wealth of new technology made available in the last decades, in fields as varied as proteomics, genomics and bioinformatics, has allowed the scientific community to study natural products and their potential uses more easily and thoroughly [2,3,7].

Sweet cherry (*Prunus avium* L.), a member of the family Rosaceae, genus *Prunus* and subgenus *Cerasus* [8], is one of the most appreciated fruits worldwide. Its biggest producer is Turkey, followed by the United States of America, the Islamic Republic of Iran and Italy [9]. Most of the sweet cherry production is for fresh consumption, with approximately 40% being processed as brined, canned, frozen, dried or juiced [8]. Sweet cherries are very nutritious fruits with their proposed health benefits mostly stemming from their high levels of phytochemicals, moderate levels of carbohydrates, and low amounts of calories [10]. Nevertheless, their precise nutritional composition is highly dependent on external influences and processing [10].

The most well-documented biological effects of sweet cherry extract encompass its antioxidant and anti-inflammatory properties [8,11,12]. The continuous increase of oxidative stress (OS) and chronic inflammation are important driving forces in the carcinogenic process, promoting cancer onset, progression and aggressiveness [13,14,15,16], which per se justifies envisaging the anti-cancer role of sweet cherry. However, more recently, research from our research group and others has started to unveil the remarkable effects of sweet cherries against many of the established hallmarks of cancer.

The first part of the present review recalls the chemical composition of the sweet cherry, its main nutrients and bioactive compounds, while also discussing their bioaccessibility and bioavailability. The remaining topics summarize the current knowledge concerning the protective effect of sweet cherries against OS, chronic inflammation, deregulated cell proliferation and apoptosis, invasion and metastization. The role of sweet cherry extracts in the suppression of metabolic reprogramming, a more recent cancer hallmark, is also revised. Overall, this review discusses the anticarcinogenic potential of sweet cherries and highlights their possible use in cancer treatment.

## 2. Nutrients, Phytochemical Composition and Bioactive Compounds

The chemical composition of sweet cherries depends on several factors, including cultivar, ripening stage, agricultural practices and edaphoclimatic conditions [10]. Sweet cherries are mainly composed of water, but they are also rich in several nutrients, such as carbohydrates (sugars and fiber), fatty and organic acids, amino acids, vitamins, minerals and phytochemicals such as melatonin, carotenoids, phenolic acids (hydroxycinnamic derivatives) and flavonoids (anthocyanins, flavanols and flavan-3-ols) [10], as depicted in Table 1.

### 2.1. Macronutrients

The three main classes of macronutrients present in sweet cherries are carbohydrates, proteins and fat (Table 1). These macronutrients comprise the essential dietary building blocks and have major roles in human body function, namely in energy production, growth and development, with frequent interplay in their metabolic and biochemical pathways [17].

Sweet cherries are mostly composed of water (>80%), presenting a moderate amount of carbohydrates (≈16%), especially sugars (sucrose, glucose, fructose, maltose and galactose) and fiber (2.1%) [18]. They also present reduced levels of fat (0.2%), particularly saturated fat, and are cholesterol-free and low in calories. Several amino acids have been additionally detected in sweet cherries, with aspartic acid being the most abundant [19]).

### 2.2. Micronutrients

Although required in minute amounts compared with macronutrients, adequate micronutrient levels, specifically of vitamins and minerals, are critical for adequate metabolic function [20].

Sweet cherry fruits are a nutrient-dense food with significant amounts of important micronutrients and are considered a source of vitamins and minerals. They are especially enriched in vitamin C and potassium, phosphorus, calcium and magnesium (Table 1) [12,18].

### 2.3. Phytochemical Composition and Bioactive Compounds

Sweet cherries are known as a relevant source of phenolic compounds (558 mg/100 g, Table 1) which include significant amounts of anthocyanins (1734 mg/100 g), other flavonoids (396 mg/100 g) and phenolic acids (162 mg/100 g). Thus, the major phenolic compounds found in sweet cherries are anthocyanins (Table 1) which have been indicated as their main bioactive compounds. The phenolic acids most abundant in sweet cherries are mainly hydroxycinnamic acids. The chemical characterization of sweet cherries has shown that neochlorogenic and *p*-coumaroylquinic acids are the predominant hydroxycinnamates (Table 1, [10,12,18,19]). Sweet cherries also contain interesting amounts of melatonin (≈1586 ng/100 g, Table 1) which is a neurohormone produced by the pineal gland, responsible for the regulation of several biological and physiologic processes in the human body, namely the regulation of circadian rhythm and, consequently, the alleviation of sleep disorders [21]. Overall, it is the phytochemical composition that mainly determines the biological importance of sweet cherries.

**Table 1:** Main nutrients, phytochemicals and bioactive compounds found in sweet cherries.

Compounds		Amount (per 100 g of Sweet Cherry)	Reference
Water		82.25 g	[22]
Macronutrients	Protein	1.06 g	[22]
	Fat (total lipids)	0.20 g	[22]
	Carbohydrates	16.01 g	[22]
Fatty acids	Total saturated	0.04 g	[22]
	Total monounsaturated	0.05 g	[22]
	Total polyunsaturated	0.05 g	[22]
Fiber (total dietary)		2.10 g	[22]
Amino acids	Tryptophan	9.00 mg	[22]
	Threonine	22.00 mg	[22]
	Isoleucine	20.00 mg	[22]
	Leucine	30.00 mg	[22]
	Lysine	32.00 mg	[22]
	Methionine	10.00 mg	[22]
	Cystine	10.00 mg	[22]
	Phenylalanine	24.00 mg	[22]
	Tyrosine	14.00 mg	[22]
	Valine	24.00 mg	[22]
	Arginine	18.00 mg	[22]
	Histidine	15.00 mg	[22]
	Alanine	26.00 mg	[22]
	Aspartic acid	56.90 mg	[22]
	Glutamic acid	83.00 mg	[22]
	Glycine	23.00 mg	[22]
	Proline	39.00 mg	[22]
	Serine	30.00 mg	[22]
Sugars	Sugars (total)	12.82 g	[22]
	Sucrose	0.15 g	[22]
	Glucose	6.59 g	[22]
	Fructose	5.37 g	[22]
	Maltose	0.12 g	[22]
	Galactose	0.59 g	[22]
Micronutrients: Minerals	Calcium	13.00 mg	[22]
	Iron	0.36 mg	[22]
	Magnesium	11.00 mg	[22]
	Phosphorus	21.00 mg	[22]
	Potassium	222.00 mg	[22]
	Zinc	0.07 mg	[22]
	Copper	0.06 mg	[22]
	Manganese	0.07 mg	[22]
	Fluoride	0.01 mg	[22]
Micronutrients: Vitamins	Vitamin C	7.00 mg	[22]
	Thiamine (Vitamin B1)	0.03 mg	[22]
	Riboflavin (Vitamin B2)	0.03 mg	[22]
	Niacin (Vitamin B3)	0.15 mg	[22]
	Pantothenic acid (Vitamin B5)	0.20 mg	[22]
	Vitamin B6	0.05 mg	[22]
	Folate (Vitamin B9)	0.01 mg	[22]
	Choline (Vitamin B4)	6.10 mg	[22]
	Vitamin A	0.01 mg	[22]
	Vitamin E	0.07 mg	[22]
	Vitamin K	0.01 mg	[22]
Phenolic Compounds	3-*O*-Caffeoylquinic acid	83.00 mg	[18]
	Catechin hexoside	168.00 mg	[18]
	Gallic acid	0.51 mg	[19]
	*p*-Coumaric acid	2.28 mg	[19]
	Rutin	10.66 mg	[19]
	Chlorogenic acid	2.95 mg	[19]
	Cyanidin-3-*O*-glycoside	22.03 mg	[23]
	Quercetin-3-4′-di-*O*-glycoside	24.61 mg	[23]
	Epicatechin	1.51 mg	[19]
	cis-*p*-Coumaroylquinic acid	56.00 mg	[18]
	trans-*p*-Coumaroylquinic acid	23.00 mg	[18]
	Taxifolin-*O*-deoxyhexosylhexoside	66.00 mg	[18]
	Taxifolin-*O*-hexoside	13.00 mg	[18]
	Quercetin-*O*-rutinoside-*O*-hexoside	42.00 mg	[18]
	Naringenin-*O*-hexoside	17.00 mg	[18]
	Dihydrowogonin 7-*O*-glucoside/sakuranetin 5-*O*-glucoside	62.00 mg	[18]
	Phenolic acids	162.00 mg	[18]
	Flavonoids (non-anthocyanins)	396.00 mg	[18]
	Total phenolic compounds	558.00 mg	[18]
	Cyanidin-3-*O*-glucoside	219.00 mg	[18]
	Cyanidin-3-*O*-rutinoside	1450.00 mg	[18]
	Peonidin-3-*O*-glucoside	64.00 mg	[18]
	Anthocyanins	1734.00 mg	[18]
Other Bioactive Phytochemicals (Carotenoids and Melatonin)	β-Carotene	38.00 µg	[22]
	Lutein + zeaxanthin	85.00 µg	[22]
	Melatonin	1.60 µg	[21]

## 3. Bioaccessibility and Bioavailability of Bioactive Compounds

Before interfering with biological activities in the human body, the bioactive compounds contained in sweet cherries (Table 1, [18,23,24]) should become bioaccessible and bioavailable. The concept of bioaccessibility is related to the quantity of a specific compound released from a matrix, which will be available for absorption after undergoing digestion [25]. Bioavailability corresponds to the amount of compound that can achieve systemic circulation and exert an effect after tissue distribution [25]. The mechanisms associated with the transport and metabolism of the distinct classes of bioactive compounds are impossible to reproduce completely but in vitro models simulating the digestive process and the use of cell lines morphologic and functionally similar to the lining of the small intestine, as the human colon carcinoma derived Caco-2 cell line, have been widely used [26,27]. These models are simple predictive instruments providing valuable information about the bioaccessibility and bioavailability of bioactive compounds present in plant and fruit extracts.

Several studies with distinct methodological approaches have been performed to access the bioaccessibility and bioavailability of sweet cherry compounds, demonstrating that they circulate in the human blood as intact or metabolized conjugates. Martini et al. studied the bioaccessibility of phenolic bioactive compounds of two different cherry cultivars, Celeste and Durone Nero I after in vitro gastrointestinal digestion with fluids simulating salivary, gastric and intestinal digestion [24]. A remarkable decrease of total and individual phenolic compounds was observed after the digestion process, with only 39.7% and 29.9% of total phenolic compounds becoming bioaccessible. Moreover, the authors identified the hydroxycinnamic acids (coumaroylquinic, feruloylquinic and caffeoylquinic acid), and some flavanols, as the compounds most resistant to gastrointestinal conditions and easily released from the cherry’s matrices and so with the highest bioaccessibility, resulting in higher antioxidant and anti-proliferative activities. Interestingly, isomers of caffeoylquinic and coumaroylquinic acids were also found after the digestion process. In the Nero I cherries cultivar, anthocyanins also appear with elevated bioaccessibility, with rutinoside derivatives, such as cyanidin-3-*O*-rutinoside, remaining more stable after in vitro digestion than their glycosidic forms [24].

A study performed by Duarte AP’s research team evaluated the bioaccessibility of the bioactive phenolic compounds present in the Saco cherries cultivar from the Fundão region of Portugal [23]. A simulated digestive process using salivary, gastric, duodenal and bile fluids was applied, and the compounds resultant from the cherries’ digestion were analyzed by high-performance liquid chromatography (HPLC). As expected, the concentration of several bioactive phenolic compounds, such as gallic acid, *p*-coumaric acid, rutin, chlorogenic acid, cyanidin-3-*O*-glycoside and quercetin-3-4′-di-*O*-glycoside was diminished at the end of the digestion process relative to the original samples. Importantly, the concentration of some of these phenolic compounds, namely quercetin, and gallic or *p*-coumaric acids, increased during the digestion procedure, which seems to be related with their conversion to other compounds, for example, the hydrolysis of their heterosidic forms. This study also investigated the bioavailability of the bioactive compounds before and after the digestion process by analyzing their absorption through the Caco-2 cell barrier. The polyphenols identified after the digestive process were all able to be absorbed by the cell barrier, although in decreased levels, becoming bioavailable. In contrast, in the cherry extract not subjected to digestion, only quercetin-3,4′-di-*O*-glycoside could cross the cell barrier and become bioavailable. Noteworthy, the extract’s antioxidant capacity disappeared after absorption by the cellular monolayer, which is in line with the decrease of total and individual phenolic compounds. Furthermore, cell monolayer integrity was analyzed, and the extracts that underwent the digestive process did not affect cell integrity, whereas the original extracts (not undergoing digestion) modified cellular integrity and increased their permeability [23]. These results highlight the indispensable function of digestion in determining the bioaccessibility and bioavailability of bioactive compounds and minimizing the interference with the integrity and permeability of intestinal cells.

In a previous study published in 2008, Fazzari M. et al. mimicked the gastric digestion of phenolic compounds from five frozen sweet cherry cultivars (Bing, Lapins, Skeena, Staccato, and Sweetheart) using pancreatin digestion [28]. Samples were dialyzed using a membrane to simulate the intestinal wall, and serum- and colon-accessible fractions of total phenols and anthocyanins were assessed using spectrophotometric and HPLC analysis. At the end of the process, Skeena, Lapins, and Sweetheart cultivars contained higher levels of total phenolic compounds and anthocyanins in both fractions, which resembled the higher content of these compounds in the original non-digested samples. Generally, the percentage of total phenolic and anthocyanin compounds on the serum-available side was lower than in the colon-available fraction. The ripening stages of these fruits also contributed to the bioaccessibility of the bioactive compounds. The authors found that immature cherries from Bing and Lapins cultivars contained a higher % of total phenolics, in the serum-available fractions, than mature or overmature cherries. Moreover, the % recovery of neochlorogenic and *p*-coumaroylquinic acids in those fractions was also usually higher for the immature cherries [28].

Bioavailability analysis of sweet cherry compounds has also been performed in fruit derivatives, namely wine [29]. The digestion methods applied included gastric, pancreatic and bile salts solutions followed by a dialysis process using a cellulose membrane. Total phenolic contents of cherry wine decreased after post-gastric digestion, accompanied by a decrease in the extract’s antioxidant capacity. The major phenolic compound observed in all phases was gallic acid, whereas quercetin was not detected after the digestion process. Caffeic acid and *p*-coumaric acid seemed to be more available in the serum fraction than in the colon fraction, whereas the opposite was observed for rutin [29].

Despite the difficulty in making a reliable comparison between the different studies due to variations in the approaches used for stimulating the in vitro digestion and the distinct samples of sweet cherry and cultivars used, some commonalities could be observed. In general, a decrease in the total bioactive phenolic compounds was observed after the in vitro digestion process. This decrease may be correlated with pH changes in the digestion medium and the activity of gastrointestinal digestive enzymes since they facilitate the release of phenolic compounds from the matrix. In addition, the phenolic structure can lose stability and suffer hydrolysis. Flavanols (e.g., quercetin acids) and hydroxycinnamic acids (e.g., coumaric acids) seem to be the bioactive compounds with the highest bioaccessibility in the different cherry cultivars evaluated. Nevertheless, standardization of the methods used by the different authors will be of paramount importance to establish the bioaccessibility and bioavailability of the bioactive compounds present in sweet cherries. Moreover, in vivo and in vitro approaches using co-cultures models that more realistically mimic the intestinal epithelium should be developed.

## 4. Sweet Cherries and the Hallmarks of Cancer

### 4.1. Oxidative Stress

The production of reactive oxygen species (ROS), hydrogen peroxide (H_2_O_2_), hydroxyl radicals and reactive nitrogen species is essential for cell function and tissue homeostasis [30,31]. However, the abnormal accumulation of these molecules causes OS and subsequent cell damage. The continuous increase of OS, and moderate amounts of ROS, have been associated with tumor onset, growth, progression and aggressiveness [13,14]. On the other hand, induction of programmed tumor cell death by extreme increase of OS has been recently exploited as an anti-cancer therapy [32,33]. Nevertheless, the antioxidant properties of natural bioactive compounds have been shown to be useful in counteracting the moderate levels of OS and the downstream processes that promote cancer development and progression.

In vitro antioxidant assays, such as 2,2’-azino-bis(3-ethylbenzothiazoline-6-sulfonic acid) (ABTS), 2,2-diphenyl-1-picrylhydrazyl (DPPH), ferric reducing ability of plasma (FRAP), oxygen radical absorbance capacity (ORAC) and nitric oxide (NO) assays, have demonstrated that sweet cherries display a high capacity to capture free radicals [34,35,36,37,38,39,40,41,42,43]. Moreover, studies reported that sweet cherry extracts inhibit lipid peroxidation [44,45] and the oxidation of human low-density lipoprotein (LDL) [40,46,47] and liposomes [47,48]. Considering that lipids are highly susceptible to oxidation and a major cause for the increase of OS, these findings corroborate the antioxidant effect of sweet cherries.

The great antioxidant capacity of sweet cherries is influenced by the pattern of bioactive compounds they contain (Table 1) which is slightly distinct for each variety and depends on several factors, such as the cultivar location, climate and harvest time [34,35,36,38,41,43,46,49]. In general, sweet cherries with higher total phenolic content display higher antioxidant capacity [35,36,37,38,40,41,49]. Moreover, it was demonstrated that among all phenolic compounds, anthocyanins, more precisely, cyanidin-3-*O*-rutinoside and cyanidin-3-*O*-glucoside, are the important contributors to the high antioxidant capacity of sweet cherries [38,40]. A study with cyanidin-3-*O*-glucoside showed that this anthocyanin displays a protective effect on DNA cleavage, a concentration-dependent free radical scavenging activity and a significant capacity to inhibit xanthine oxidase activity [50]. In an OS rat model, cyanidin-3-*O*-glucoside significantly suppressed liver damage caused by hepatic ischemia-reperfusion [51]. Besides anthocyanins, *p*-coumaroylquinic acid [40,41] and other flavanols and flavonoids are also important antioxidant phenolic compounds found in sweet cherries [41]. In addition, this interesting fruit contains vitamin A, C and E, carotenoids and melatonin, which are also powerful antioxidant molecules [52,53,54,55].

The majority of existent studies employed biochemical assays to demonstrate the antioxidant activity of sweet cherry extracts. However, in vitro studies with different cell cancer models also showed the biological potential of sweet cherry extracts in suppressing OS (Table 2, [38,40,42,45,49,52,56]). In human hepatocellular carcinoma HepG2 cells, sweet cherry extract reduced OS, with a differential response depending on the fruit variety and its amount of phenolic content [38]. Both sweet cherry extracts with low and high phenolic content showed an antioxidant effect and were capable of reducing OS. However, for sweet cherry extracts with lower phenolic content, the effects were only seen for low concentrations of the extracts. At higher extract concentrations, the increased concentrations of glucose and fructose relative to phenolic compounds were linked with the increased levels of ROS. Concerning sweet cherry extracts with higher quantities of phenolic compounds, the increase in OS with the increase of extract concentration was not observable [38].

In Caco-2 cells, the antioxidant effect of sweet cherries was related to the anthocyanin content [40,42,52]. Sweet cherry extracts with higher total phenolic content, particularly, higher anthocyanin concentration, displayed a protective effect against oxidative damage caused by OS inducers, such as tert-butyl hydroperoxide (*t*-BHP) and H_2_O_2_ [42,52]. Furthermore, the antioxidant protection was more effective in Caco-2 cells co-treated with the OS inducer and sweet cherry extracts highly enriched in anthocyanins, compared with cells treated with the extract alone. This difference may be explained by the fact that anthocyanins are modestly absorbed by Caco-2 cells, despite having a powerful effect as scavengers of extracellular free radicals [40,42,52]. In contrast, no differences were found between the treatment with the fruit extract alone and the co-treatment with the OS inducer for the sweet cherry extract with lower content of phenolic compounds [40]. The phenolic compounds that are present in these sweet cherry extracts, namely chlorogenic acid, catechin and rutin, are absorbed by Caco-2 cells, which justifies the different findings obtained. Moreover, these findings indicate that the phenolic compounds absorbed by Caco-2 cells mediate the intracellular antioxidant response.

**Figure 1:**
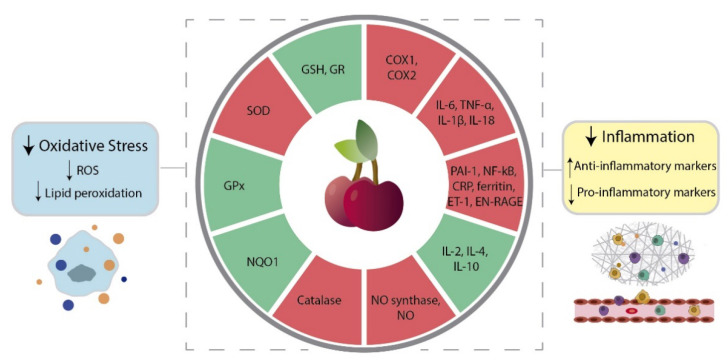
Sweet cherry modulation of oxidative stress and inflammation. Sweet cherry extracts reduce oxidative stress by decreasing the production of reactive oxygen species (ROS), namely nitric oxide (NO) and lipid peroxidation. This was accompanied by the decreased activity of nitric oxide (NO) synthase, up-regulation of glutathione (GSH), and altered expression of several enzymes involved in the antioxidant defense, such as glutathione reductase (GR), glutathione peroxidase (GPx), NAD(P)H quinone oxidoreductase (NQO1), catalase, and superoxide dismutase (SOD). The anti-inflammatory effect of sweet cherries is achieved by both inducing anti-inflammatory markers whereas inhibiting the pro-inflammatory ones. Identified targets include the anti-inflammatory (interleukin (IL)-2, -4 and -10) and pro-inflammatory (IL-6, -1β and -18) cytokines tumor necrosis factor (TNF)-α, cyclooxygenase (COX) 1 and COX2, nuclear factor kappa-light-chain-enhancer of activated B cells (NF-κB), C-reactive protein (CRP), epidermal growth factor (EGF), endothelin-1 (ET-1), extracellular newly identified receptor for advanced glycation end-products binding protein (EN-RAGE), ferritin and plasminogen activator inhibitor-1 (PAI-1). Green and red circle sections mean activation and inhibition, respectively.

The mechanisms behind the antioxidant activity of sweet cherry extract have also started being discovered (Figure 1). In Caco-2 cells, sweet cherry extracts enriched in polyphenols restored the reduced glutathione/oxidized glutathione (GSH/GSSG) ratio [42]. Similarly, in human neuroblastoma SH-SY5Y cells, the sweet cherry extracts with the highest content of anthocyanins were the most effective in protecting against OS induced by H_2_O_2_, by reducing intracellular ROS levels and increasing GSH [49]. In addition, this study reported that the sweet cherry extracts with the highest antioxidant effect also increased the levels of two important antioxidant enzymes, namely, glutathione reductase (GR) and NAD(P)H quinone oxidoreductase (NQO1) [49]. However, further research is needed to fully ascertain the capability of sweet cherry extract in modulating the activity of enzymes of the antioxidant defense system. It was demonstrated in human neuroblastoma SK-N-MC cells that a 2 h pre-incubation with a sweet cherry extract enriched in phenolic compounds reduces the accumulation of intracellular ROS upon injury with H_2_O_2_ [42]. These effects were not observed when the pre-incubation time was 24 h, which indicates that the extract’s antioxidant action occurs through the direct scavenging of ROS, and not implicating the modulation of other endogenous mechanisms [42]. Also, in human prostate cancer LNCaP cells, sweet cherry extract had a protective effect against oxidative damage and lipid peroxidation [45]. However, the extract failed to influence the activity of superoxide dismutase (SOD) and glutathione peroxidase (GPx) [45]. Even considering the limitations discussed in Section 3 related to the bioavailability and bioaccessibility of bioactive compounds, in vivo evidence of the antioxidant capacity of sweet cherries remains compelling (Table 2). In Wistar rats fed a high fructose diet it was demonstrated that freeze-dried sweet cherries increased the levels of GR and GPx, and inhibited lipid peroxidation and the activity of catalase and SOD [57]. This diminished activity of catalase and SOD may be explained by the fact that the intake of antioxidants through diet reduces the need to activate endogenous antioxidant enzymes.

The beneficial effects of sweet cherry consumption have also been evaluated in humans. Prior et al. demonstrated that the daily consumption of 280 g of sweet cherries for 6 consecutive days increased plasma lipophilic and hydrophilic antioxidant capacity [58]. Another study showed that consuming 200 g of sweet cherries twice a day for 3 days increased the urinary antioxidant capacity levels, presumably due to the antioxidant role of tryptophan [59]. A similar study in 10 healthy women demonstrated that the daily consumption of 280 g fruit for 6 days after overnight fast increased lipophilic ORAC and decreased FRAP levels [60]. The decreased levels of FRAP are not surprising considering that in this study, sweet cherry consumption decreased urate levels, the largest contributor to plasma hydrophilic antioxidant capacity [61,62].

### 4.2. Inflammation

Inflammation is an essential and complex physiological response to tissue damage caused by several acute causes such as physical injury, infection or exposure to toxins [63]. However, when inflammation becomes chronic, this biological response becomes harmful and may lead to the development of chronic diseases [15,16,63]. Chronic inflammation has been indicated as one of the major causes involved in cancer development and progression [15,16].

Among a panoply of other benefits and cytoprotective effects, natural bioactive compounds have also been shown to display anti-inflammatory properties, being useful tools to counteract chronic inflammation [63]. A mark of the inflammatory response is the dramatic increase of prostaglandin levels due to the activation of cyclooxygenases (COX) [64]. In vitro biochemical studies showed that whole sweet cherry extracts, as well as extracted anthocyanins, inhibited COX1 and COX2 activity (Figure 1), with a more noticeable effect for COX2 [44,48,65]. This is an impactful outcome envisaging an anti-cancer role for sweet cherries, as COX2 is the most important source of prostaglandins in cancer cases [66,67].

In biological models, the anti-inflammatory effect of sweet cherry extracts, anthocyanins and their metabolites (Table 2) were shown to be a consequence of the regulation of other pro- and anti-inflammatory markers, such as interleukin (IL)-6 and IL-10, respectively (Figure 1, [56,57,60,62,65,68,69,70,71,72,73]). An in vitro study using human acute monocytic leukemia THP-1 cells treated with monosodium urate (MSU) crystals, the main causative agent for the acute inflammatory response in gout, further detailed the anti-inflammatory effect of sweet cherries. In the presence of sweet cherry extract, the levels of the pro-inflammatory protein IL-1β were reduced, concomitantly with the inhibition of the crystals’ phagocytosis. In acute pain episodes, IL-1β is released in response to MSU phagocytosis, which led the authors to suggest that sweet cherry extract can reduce gout-associated inflammation [56].

The anti-inflammatory effects of sweet cherries were also demonstrated in vivo (Table 2). The effect of a sweet cherry-based beverage was evaluated in Wistar rats and ringdoves birds of different ages [70]. This study showed that the sweet cherry-based beverage modulated IL-1β, IL-4 and IL-2 levels, in both young and old animals, with species- and age-dependent effects, and under the influence of the circadian rhythm. For example, the downregulation of IL-1β was not observed in old rats in the afternoon, in old birds at dawn, and in young birds at the acrophase (acrophase of the melatonin rhythm). Similarly, differences were also found considering the upregulation of the anti-inflammatory IL-2. IL-2 levels increased in young birds only during the acrophase and in old birds through dawn and acrophase. In rats, the upregulation of IL-2 dissipated in the afternoon in old animals and in the acrophase in both young and old. Moreover, the sweet cherry-based beverage also downregulated the levels of tumor necrosis factor (TNF)-α, with effects observed at dawn in old rats and young birds, and during the afternoon and at the acrophase in old birds [70].

In Wistar rats fed with a high-fructose diet, the co-administration of freeze-dried sweet cherry increased the levels of IL-10 and decreased C-reactive protein (CRP), another well-known inflammatory marker [57].

The contribution of specific phenolic components to the sweet cherry’s anti-inflammatory role was also analyzed [71,74]. In obese diabetic mice, diet supplementation with a non-anthocyanin phenolic sweet cherry powder lowered IL-6 to levels similar to that of lean mice [71]. Moreover, in diet-induced obese mice the administration of cyanidin-3-(2^G^-glucosylrutinoside), cyanidin-3-rutinoside and pelargonidin-3-glucoside extracted from sweet cherries decreased the levels of IL-6, TNF-α, inducible NO synthase, and nuclear factor kappa-light-chain-enhancer of activated B cells (NF-κB, Figure 1) [74].

The anti-inflammatory effect of the direct intake of sweet cherries has also been reported in humans. Daily consumption of 280 g sweet cherry for 28 days decreased the levels of CRP and NO in 20 healthy subjects [60]. However, 28 days postintervention CRP and NO levels were partially reverted to their initial state.

Another study reported the effects of the daily consumption of 280 g of sweet cherry for 28 days in 18 healthy patients [62]. CRP, epidermal growth factor (EGF), endothelin-1 (ET-1), extracellular newly identified receptor for advanced glycation end-products binding protein (EN-RAGE), ferritin, IL-18 and plasminogen activator inhibitor-1 (PAI-1) levels decreased (Figure 1) whereas the expression of IL-1 receptor antagonist was increased. Moreover, patients were analyzed 28 days after treatment to understand if the anti-inflammatory effects of sweet cherry consumption were maintained. In the postintervention period, ferritin levels continued to decrease, and the low levels of CRP were maintained. However, the levels of the other biomarkers were completely or partially reversed [62].

Very interestingly, short time effects after sweet cherry consumption have been reported. In 10 healthy women, the daily consumption of 280 g sweet cherries for 6 days slightly decreased CRP, but 3 h after consumption were enough to observe a diminution of NO levels [72].

### 4.3. Cell Death and Proliferation

The strict control of cell proliferation and apoptosis, and an accurate balance between these biological processes are essential to maintain cell number and tissues’ homeostasis [75]. Also, it is unquestionable that any disturbance in this equilibrium can alter normal tissue architecture and function, likely contributing to cancer development. In fact, sustained proliferative signaling and resistance to cell death are well-established cancer hallmarks, involved in tumor onset, progression and aggressiveness. The pro-survival and high proliferative features of cancer cells are driven by the hyper-activation of survival signaling pathways whereas signal transduction pathways involved in the suppression of cell proliferation and apoptosis induction are inhibited [75,76].

The last years have witnessed the confirmation that sweet cherry extract can have a role in influencing the survival and apoptosis of cancer cells (Table 2). These properties are uniquely determined by the extract’s chemical composition (Table 1) and its enrichment in specific bioactive components, which, as discussed in Section 3, depends on several factors, namely the cultivar place and harvest time [19,24,40]. In vitro and in vivo studies demonstrated that polyphenols are the main responsible for the anti-proliferative and pro-apoptotic effects among the sweet cherries’ bioactive compounds [18,19,24,40,45,77,78,79,80,81]. Sweet cherry extracts rich in polyphenols inhibited the viability of human prostate non-neoplastic PNT1A cells, and human lung A549, cervix HeLa, brain SK-B-NE (2)-C, SH-SY5Y and prostate cancer LNCaP cells in a concentration-dependent manner [19,45]. Moreover, it was shown that HeLa cells were the most sensitive to the effect of sweet cherry extract [19]. The inhibitory effect on cell viability was also reported in human castrate-resistant prostate cancer PC3 and gastric carcinoma MKN45 cells, though independently on the concentration of sweet cherry extract used [40,45].

The presence of sweet cherry extract showed to reduce the proliferative rate of several types of human cancer cell lines (Table 2). In human colon carcinoma cell lines (HCT-15, HT29 and SW480 cells) extract concentrations between ~0.074 and 13.8 mg/mL reduced cell proliferation by 50% [18,24,40,77,78]. The antiproliferative effect observed in HT29 cells was time and concentration-dependent [77,78] and associated with cell cycle arrest in G1/G0 phase [77]. Furthermore, in vitro digestion of the sweet cherry extracts enriched in phenolic compounds halved the extract concentration needed to decrease SW480 cells proliferation by 50% [24], suggesting that other bioactive compounds beyond anthocyanins may be responsible for the observed effect.

**Figure 2:**
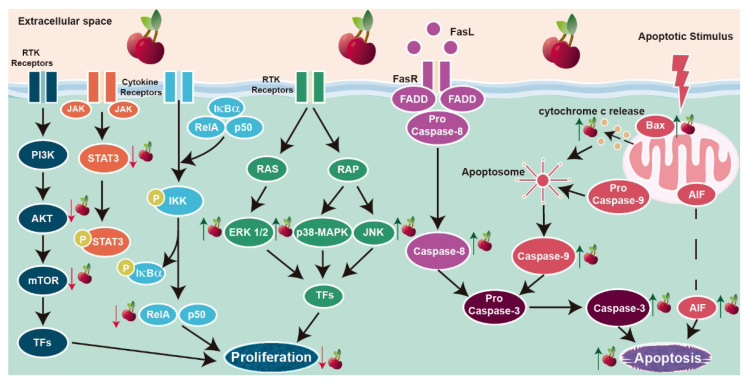
Sweet cherries effects in modulating the intracellular signaling that governs cancer cell proliferation and apoptosis. Regardless of anthocyanins or proanthocyanins enrichment, sweet cherry extracts reduced cell proliferation and induced apoptosis with altered expression and/or activity of several molecular targets. However, extracts enriched in anthocyanins or proanthocyanins can influence specific molecular targets (see text for details). Overall, mechanistically, the phosphoinositide 3-kinase (PI3K) pathway was inhibited with the mechanistic target of rapamycin (mTOR) and AKT as targets. Cytokine receptors signaling could also be influenced by sweet cherry extract, namely by the modulation of signal transducer and activator of transcription (STAT) 3 and nuclear factor kappa-light-chain-enhancer of activated B cells (NF-κB or RelA/p50). In the mitogen-activated protein kinase (MAPK) pathway, altered activity of p38-MAPK, extracellular signal-regulated kinase (ERK) 1/2, and c-Jun N-terminal kinase (JNK) was reported. Concerning apoptosis, the sweet cherry extract enriched in anthocyanins influenced both the extrinsic (caspase-8) and intrinsic (B-cell lymphoma 2-associated X protein (Bax), apoptosis-inducing factor (AIF), cytochrome c release, caspase-9) pathways, culminating in the activation of caspase-3. Whole sweet cherry extract or extract enriched in proanthocyanins activated only the intrinsic pathway (Bax, AIF, caspase-9 and -3, and cytochrome c release). Green and red arrows mean up- and down-regulation of expression and/or activity, respectively. Legend: FADD, Fas-associated protein with death domain; FasL, Fas ligand; FasR, Fas receptor; IκBα, nuclear factor of kappa light polypeptide gene enhancer in B-cells inhibitor, α; IKK, IκB kinase; JAK, Janus Kinase; RAP, Ras proximate; RTK, Receptor tyrosine kinase; TFs, Transcription factors.

Interestingly, an important anti-proliferative effect of sweet cherry extracts was observed in breast cancer cells, with no toxicity to the non-neoplastic MCF-10A breast cells [79]. Sweet cherry juice whole extract inhibited the growth of human breast MDA-MB-231 and BT-474 cancer cell lines with the same potency, presenting higher capacity inhibiting the growth of the triple-negative MDA-MB-453 cells [79]. Moreover, a sweet cherry juice extract enriched in anthocyanins or proanthocyanins preferentially inhibited MDA-MB-453 cell proliferation [79]. These are very interesting findings considering the aggressiveness of triple-negative breast cancer and the lack of approaches to efficiently manage this human neoplasia. Also, they were accompanied by the disclosure of the putative underlying mechanisms (Figure 2). They include the PI3K/AKT survival pathway that is overactivated in cancer cases and associated with cell growth, proliferation and survival in several types of cancer [82]. Experiments using three different types of extracts, whole sweet cherry extract or extracts enriched in anthocyanins or proanthocyanins, showed that all decreased AKT mRNA levels [79]. However, only anthocyanins-enriched extract decreased the levels of the active phosphorylated AKT (p-AKT, Figure 2) [80]. The downstream targets of PI3K/AKT signaling were also modulated by the sweet cherry extract. Although the mRNA levels of the mechanistic target of rapamycin (mTOR) were decreased, its protein expression was increased in response to whole sweet cherry extract and anthocyanins- or proanthocyanins-enriched extracts [79]. Moreover, the levels of phosphorylated mTOR (p-mTOR) decreased (Figure 2), inducing a decrease in the p-mTOR/mTOR protein ratio (Figure 2) and the inhibition of this signaling pathway [79]. Because of its overactivation in cancer, the PI3K/AKT pathway has been considered a valuable therapeutic target [82]. Therefore, the inhibition of this signaling pathway by the sweet cherry extract reinforces the importance of continuing to study this fruit as an anticarcinogenic and its potential use in cancer treatment.

The activity of sweet cherry extract was also shown to influence the p38-mitogen-activated protein kinase (p38-MAPK) signaling pathway (Figure 2). In MDA-MD-453 cells, sweet cherry extract increased the p-p-38-MAPK/p38-MAPK protein ratio and enhanced the expression levels of the extracellular signal-regulated kinase (ERK) 1/2, and c-Jun N-terminal kinase (JNK) phosphorylated forms [80]. However, the p-ERK1/2/full ERK1/2 ratio was not altered. Nevertheless, treatment with U0126, an inhibitor of the ERK1/2 signaling pathway, abrogated the downregulation of p-AKT induced by the sweet cherry extract enriched in anthocyanins [80]. This finding is quite interesting because it demonstrates that the effect of sweet cherry extract negatively regulating the PI3K/AKT pathway can be modulated by the ERK1/2 pathway. The cross-talk between PI3K/AKT and MAPK pathways, namely the relationship of ERK1/2 activity with p-AKT levels, also was found in vascular endothelial cells treated with euscaphic and tormentic acid [83]. Indeed, the interconnection of these pathways has been described in different physiological conditions [84,85]. Further research is needed to clarify how it can be a target of natural bioactive compounds in cancer cells.

Studies concerning the effect of sweet cherry extracts in apoptosis are scarce, being limited to human breast and prostate cells [45,79,80]. Sweet cherry extracts induced apoptosis in the breast cancer MDA-MB-453 cell model by controlling the expression and activity of cell survival regulators and signaling pathways [79,80]. Similarly to what was described for cell proliferation, the effect of sweet cherry extracts on apoptosis targeted the PI3K/AKT and MAPK/p38-MAPK/JNK pathways, with the activation of the intrinsic and extrinsic pathways of apoptosis (Figure 2). However, differences were found related to the extract’s composition. Sweet cherry extracts enriched in anthocyanins increased the protein levels of cleaved caspase-8, apoptosis-inducing factor (AIF), cytochrome c, cleaved caspase-9 and -3, and Bax/Bcl2 protein ratio (Figure 2), which suggests that apoptosis occurs by the activation of both the intrinsic and extrinsic pathways [80]. The same study reported that sweet cherry extracts enriched in proanthocyanins or whole extract enhanced the levels of AIF, cytochrome c, cleaved caspase-9 and -3, and Bax/Bcl2 protein ratio (Figure 2), with no effect observed on caspase-8, indicating that only the intrinsic pathway was activated [80]. Independently of the specific composition of the extract under study, increased levels of cleaved poly (ADP-ribose) polymerase (PARP)-1 and enhanced cleaved-PARP-1/PARP-1 protein ratio were detected [79,80]. PARP cleavage by activated caspase-3 is a late event in the apoptotic cascade that confirms the activation of cell death by sweet cherry extracts [86]. Moreover, treating MDA-MB-453 cells with U0126 and SB203580, ERK1/2 and p-38 MAPK inhibitors, respectively, abrogated the effect of sweet cherry extracts modulating Bax and PARP-1 expression levels and reverting the activation of the intrinsic pathway [80]. Gathering all data, it is liable to assume that sweet cherry extracts enriched in anthocyanins display higher pro-apoptotic potential than the sweet cherry extracts enriched in proanthocyanins or whole extracts.

In the case of prostate cells, results from our research group demonstrated that sweet cherry extract induced apoptosis of the androgen-sensitive LNCaP cells, while having no effect on the non-neoplastic PNT1A epithelial cells [45]. However, the sweet cherry extract suppressed apoptosis in castrate-resistant PC3 cells (Table 2), which could be explained by the fact that these cells do not express p53, a crucial player mediating the apoptotic response [45]. In LNCaP cells, the higher apoptotic rate in the presence of extract was underpinned by the increased activity of caspase-3, enhanced expression of caspase-9 and augmented Bax/Bcl-2 protein ratio [45]. Therefore, on the dependency of the activation of the intrinsic pathway.

The anti-tumor activity of whole sweet cherry extract and anthocyanins- or proanthocyanins-enriched extracts was evaluated in vivo in an MDA-MB-453 cell line xenograft [81]. Oral gavage administration of all sweet cherry extracts reduced tumor growth, not affecting other mouse organs [81]. Proteomic analysis of the developing tumors showed that the sweet cherry extract enriched in anthocyanins modulated the expression of a panoply of proteins associated with the regulation of cell survival and growth, namely Ras-related proteins, ubiquitin-conjugating enzyme E2 and proliferating cell nuclear antigen [81]. Moreover, the anthocyanins-enriched extract decreased the protein levels of cell survival regulators AKT, p38-MAPK, JNK and NF-κB (Figure 2). Downregulated expression of the proliferation marker Ki-67 and the signal transducer and activator of transcription (STAT) 3, and induction of p-ERK1/2 levels were found in tumors of mice treated with all the extracts tested [81].

The discussed findings demonstrate the enormous effect of sweet cherry extracts in controlling cell survival and growth, which was also shown to have an impact on tumor development in a mouse model.

### 4.4. Invasion and Metastization

Cancer invasion and metastization arise from the convergence of several processes such as increased OS, inflammation, hyper-activation of pro-survival pathways and metabolic alterations [75,76,87], which determine the alteration of cancer cell behavior promoting their aggressiveness. Overall, these processes cause the alteration of epithelial cell morphology and cell-cell and cell-extracellular matrix adhesion [75]. Indeed, epithelial-mesenchymal transition (EMT) and the degradation of the extracellular matrix are the fundamental events leading to invasion, migration and metastization into distant organs [75,76].

In vitro studies in MDA-MB-453 cells showed that sweet cherry juice extract enriched in anthocyanins decreased invasion capacity (Table 2, [80]), which follows other findings showing that anthocyanins play an important role inhibiting tumor growth, invasion and metastization [88]. In fact, peonidin-3-*O*-glucoside, cyanidin-3-*O*-glucoside and cyanidin-3-*O*-rutinoside, the most abundant anthocyanins in sweet cherries, display anti-invasive capacities, significantly reducing the invasion of A549 cells [89,90]. Peonidin-3-*O*-glucoside and cyanidin-3-*O*-glucoside also inhibited the invasion capacity of human hepatocarcinoma SKHep-1 and Huh-7, cervical carcinoma HeLa and tongue squamous cell carcinoma SCC-4 cells in a concentration-dependent manner [91]. Nevertheless, the anti-invasive effects of sweet cherry extracts in decreasing cancer cell motility are maintained if proanthocyanins-enriched or whole extracts are used (Table 2), which indicates that other bioactive compounds in the extract contribute to that effect [80].

The mechanisms underlying the anti-invasive effect of sweet cherries are slowing being disclosed [79,80]. In the MDA-MB-453 cell model, sweet cherry extracts enriched in anthocyanins modulated the activity of PI3K/AKT/mTOR and ERK1/2/p38-MAPK/JNK signaling pathways, and also regulated phospholipase-C gamma-1 (PLCγ-1) signaling by decreasing PLCγ-1 phosphorylation [80]. This is quite relevant as PLCγ-1 has been shown to play an important role in the regulation of invasion and metastization by controlling multiple mechanisms involved in cytoskeletal alterations and cell migration [92]. The anthocyanin-enriched extract also diminished the mRNA levels of the vascular cell adhesion molecule 1 (VCAM-1) [79], an important cell adhesion molecule deeply associated with the EMT [93].

As mentioned above, the degradation of the extracellular matrix is a determinant event concerning cancer invasion and metastization, which highly depends on proteolysis events mediated by the intervention of matrix metalloproteinases (MMPs) [94]. The capacity of sweet cherries to counteract alterations in the extracellular matrix needs further investigation because discrepant findings have been described. Sweet cherry extract enriched in anthocyanins had no effect on the expression of uridylyl phosphate adenosine (uPA) and MMP-3, -9 and -10 [80]. However, isolated peonidin-3-*O*-glucoside and cyanidin-3-*O*-glucoside were capable of decreasing uPA secretion in SKHep-1, Huh-7, HeLa and SCC-4 cells, and the release of MMP-2 in SCC-4 and SKHep-1 [91].

Angiogenesis, the process of forming new blood vessels from pre-existing ones, accompanies tumor growth and invasion, with the continuous growth of the vascular network being crucial for the metastatic spread of cancer [95,96,97]. The vascular endothelial growth factor (VEGF) is the key mediator of the angiogenic process in the tumor microenvironment, also playing a role in remodeling the extracellular matrix [98,99,100]. Sweet cherry extracts enriched in anthocyanins or proanthocyanins, or the whole extract, decreased VGEF levels and/or that of the specificity protein 1, a major regulator of VEGF expression [80,101].

Information concerning the anti-metastatic effect of sweet cherry extracts in vivo is almost non-existent. However, a study with proteomic analysis in the MDA-MB-453 cells xenograft mouse model showed that a beverage of sweet cherry extract enriched in anthocyanins could modulate the expression of proteins associated with EMT cell adhesion, invasion and metastization [81]. Moreover, in rat-induced esophageal tumors [102], anthocyanins from black raspberries decreased the levels of VEGF and hypoxia inducible factor-1, the main transcription factor governing the expression of genes and proteins implicated in the angiogenic process [103].

Cell migration, invasion and metastization are critical cancer hallmarks representing a major barrier to treatment and hampering clinical outcomes [75,76]. It is noteworthy that more than 90% of cancer-related deaths are a consequence of metastasis [104]. The identification that sweet cherries or some of their specific bioactive compounds could have a role in suppressing cell motility and cancer invasiveness is a very interesting achievement. Future research in preclinical models will complete our understanding of how this fruit can modulate cancer cell invasion and metastization.

### 4.5. Metabolic Reprogramming

The establishment of metabolic reprogramming as a hallmark of cancer in 2011 [75] renewed the interest in studying cancer metabolism. Moreover, in the last decade, navigating the discovery of the metabolic peculiarities of cancer cells and their vulnerabilities widened the potential of cancer treatment targeting metabolic inhibition [105,106].

**Figure 3:**
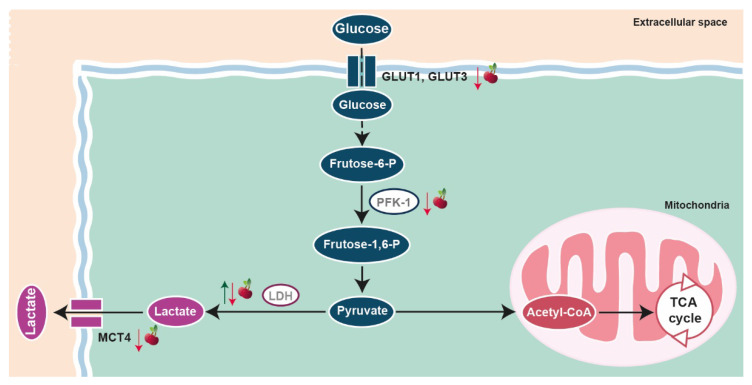
Sweet cherry actions in the regulation of glucose metabolism. Sweet cherry extract modulated glucose uptake in both neoplastic and non-neoplastic cancer cells, which was linked with the decreased expression of glucose transporters (GLUTs), GLUT1 and GLUT3. After entering the cell, glucose undergoes glycolysis with the production of pyruvate that can be converted to lactate by lactate dehydrogenase (LDH) or to acetyl-coenzyme A (Acetyl-CoA), which enters the tricarboxylic acid (TCA) cycle. In prostate cancer cells, the presence of sweet cherry extract also reduced lactate production, which was underpinned by the decreased activity of LDH and a reduced expression of the lactate exporter, monocarboxylate transporter (MCT) 4. In addition, sweet cherry extract downregulated phosphofructokinase-1 (PFK-1) expression in non-neoplastic cells, whereas increasing LDH activity. Green and red arrows mean up- and down-regulation of expression and/or activity, respectively.

The metabolic plasticity of cancer cells and their reprogramming in response to limitations imposed by the tumor microenvironment are essential in allowing the faster acquisition of energy, and sustaining tumor growth, invasion and metastization [75,106,107]. Several metabolic pathways have been shown to be exacerbated in cancer cells, which includes the glycolytic flux, known for almost a century since the pioneering studies of Otto Warburg [108], and also lipid and glutamine metabolism [106,107,109]. Recent findings from our research group showed that exposure to sweet cherry extract suppressed the glycolytic activity of the androgen-sensitive prostate cancer cells LNCaP (Table 2, [45]). The diminished glucose consumption and lactate production observed in LNCaP cells were underpinned by the reduced expression of glucose transporter (GLUT) 3 and monocarboxylate transporter (MCT) 4, and decreased activity of lactate dehydrogenase (LDH) (Figure 3). Moreover, these effects seem to be specific to this cell line that mimics a mild-aggressive stage of prostate cancer, not being followed in the non-neoplastic PNT1A cells, or in the more aggressive PC3 cell model [45].

The potential of sweet cherries and even other fruits and their bioactive compounds in modulating the metabolic reprogramming of cancer cells is a very recent research issue. Even so, other reports also have shown this influence (Table 2). Berry extract was shown to decrease glucose uptake and the expression of (GLUT) 2 in Caco-2 cells, which was mainly due to the effect of cyanidin-3-*O*-glucoside and cyanidin-3-*O*-rutinoside [110]. In line with these data, anthocyanins from grape pomace extract reduced glucose consumption, lactate production and the intracellular levels of lactate, pyruvate and glutamate in HepG2 cells [111].

In what concerns lipid metabolism, a study in a mouse model xenografted with breast cancer cells showed that a sweet cherry extract enriched in anthocyanins inhibited the expression of acetyl-CoA acetyltransferase (ACAT1) and lipase E, hormone sensitive type (Table 2, [81]). In HepG2 cells, cyanidin-3-*O*-glycoside decreased de novo lipid biosynthesis and inhibited mitochondrial F0F1-ATPase [112]. Mechanistically, the reduced activity of mitochondrial F0F1-ATPase was linked with cyanidin-3-*O*-glycoside activation of protein kinase C ζ. Importantly, the inhibition of the activity of mitochondrial F0F1-ATPase demonstrated that this anthocyanin affected the ADP/ATP ratio [112]. On the other hand, anthocyanins from grape pomace extract increased oxygen consumption and mitochondrial respiration, driving ATP synthesis [111]. Grape pomace extract is a complex mixture of different anthocyanins, which may explain the reported differences in ATP homeostasis.

It remains to be clarified if sweet cherry and other fruit extracts could have an action over energy metabolism in vivo, and if this is related to the suppression of tumor growth and metastization. Experiments in genetic cancer models or xenografts will be pivotal to shed light on this issue.

**Table 2:** Summary of the biological effects of sweet cherry extract and bioactive compounds targeting cancer hallmarks.

Hallmark of Cancer	Type of Study/Biological model		Experiment	Extract Concentration/Phenolic Content/Dose/Mass of Sweet Cherry	Time of Treatment	Effect	Reference
Oxidative Stress	In vitro	Hep2G cells	Incubation with sweet cherry extract	High phenolic content	90 min	↓ Intracellular ROS	[38]
			Pre-incubation with sweet cherry extract before H_2_O_2_ administration		24 h	↓ Intracellular ROS in a concentration dependent-manner	
			Incubation with sweet cherry extract	Low phenolic content	90 min		
			Pre-incubation with sweet cherry extract before H_2_O_2_ administration		24 h		
		Caco-2 cells	Pre-incubation with sweet cherry extract before H_2_O_2_ administration	50 GAE */mL	1 h	↓ Intracellular ROS ↓ Carbonyl proteins Restored GSH/GSSG ratio	[42]
				10 mg dry weight/mL	4 h	↓ Intracellular ROS	[40]
				25% (*v*/*v*)	24 h	↓ NO Inhibited LDH leakage	[52]
			Pre-incubation with sweet cherry extract before *t*-BHP administration	10 mg dry weight/mL	4 h	↓ Intracellular ROS	[40]
			Co-incubation with sweet cherry extract and H_2_O_2_	50 GAE/mL	1 h	↓ Intracellular ROS ↓ Carbonyl proteins Restored GSH/GSSG ratio	[42]
				10 mg dry weight/mL	4 h	↓ Intracellular ROS	[40]
			Co-incubation with sweet cherry extract and *t*-BHP	10 mg dry weight/mL			
		SH-SY5Y cells	Pre-incubation with sweet cherry extract before H_2_O_2_ administration	50 µg/mL	24 h	↓ Intracellular ROS ↑ GSH ↑ GR ↑ NQO1	[49]
		SK-N-MC cells	Pre-incubation with sweet cherry extract before H_2_O_2_ administration	1 GAE/mL	2 h	↓ Intracellular ROS	[42]
		THP-1 cells	Pre-incubation with sweet cherry extract before exposure to MSU	1.81 mg GAE/mL	3 h	↓ Intracellular ROS	[56]
				2.32 mg GAE/mL			
			Pre-incubation with MSU before treatment with sweet cherry extract	1.81 mg GAE/mL	Unknown		
				2.32 mg GAE/mL			
		LNCaP cells	Incubation with sweet cherry extract	20 µg/mL	72 h	↓ Intracellular ROS Inhibited lipid peroxidation	[45]
	In vivo	Wistar rats	High fructose-diet with freeze-dried sweet cherry	50 g/kg	12 weeks	↑ GPx Inhibited lipid peroxidation ↓ Catalase ↓ SOD	[57]
				100 g/kg		↑ GPx ↑ GR ↓ Catalase ↓ SOD	
	Human subjects	10 healthy men	Daily consumption of sweet cherries after overnight fasting	280 g	6 days	↑ Plasma lipophilic antioxidant capacity ↑ Plasma hydrophilic antioxidant capacity	[58]
		12 volunteers	Consumption of sweet cherries twice a day after lunch and diner	200 g	3 days	↑ Urinary antioxidant capacity	[59]
		10 healthy women	Daily consumption of sweet cherries after overnight fasting	280 g	6 days	↑ Lipophilic oxygen radical absorbance capacity ↓ Ferric reducing ability of plasma	[60]
Inflammation	In vitro	THP-1cells	Pre-incubation with sweet cherry extract before exposure to MSU	1.81 mg GAE/mL	3 h	↓ IL-1β Inhibited the phagocytosis of monosodium urate crystals	[56]
				2.32 mg GAE/mL			
			Pre-incubation with MSU crystals before treatment with sweet cherry extract	1.81 mg GAE/mL	Not applicable	↓ IL-1β Inhibited phagocytosis of monosodium urate crystals	
				2.32 mg GAE/mL			
	In vivo	Wistar rats	High fructose-diet with freeze-dried sweet cherry	50 g/kg	12 weeks	*↓* CRP	[57]
				100 g/kg		↓ CRP ↑ IL-10	
			Daily consumption of sweet cherry-based beverage	75,400 µg/mL	10 days	↓ IL-1β in young rats during the dawn, afternoon (18 h) and the acrophase of the melatonin rhythm ↓ IL-1β in old rats during the dawn and the acrophase of the melatonin rhythm ↓TNF-α in old rats during the dawn ↑ IL-2 in young rats during the dawn and afternoon (18 h) ↑ IL-2 in old rats during the dawn ↑ IL-4 in young rats during the dawn during the dawn, afternoon (18 h) and the acrophase of the melatonin rhythm ↑ IL-4 in old rats during the dawn during the dawn, afternoon (18 h) and the acrophase of the melatonin rhythm	[70]
		Ringdove birds	Daily consumption of sweet cherry-based beverage	75,400 µg/mL	10 days	↓ IL-1β in young birds during the dawn and the afternoon (18 h) ↓ IL-1β in old birds during the afternoon (18 h) and the acrophase of the melatonin rhythm ↓TNF-α in young birds during the dawn ↓ TNF-α in old birds during the afternoon (18 h) and the acrophase of the melatonin rhythm ↑ IL-2 in young birds during the acrophase of the melatonin rhythm ↑ IL-2 in old birds during the dawn during the dawn and the acrophase of the melatonin rhythm ↑ IL-4 in young birds during the dawn during the dawn, afternoon (18 h) and the acrophase of the melatonin rhythm ↑ IL-4 in old birds during the dawn during the dawn, afternoon (18 h) and the acrophase of the melatonin rhythm	
		Obese-diabetic mice	Diet supplemented with anthocyanin-depleted cherry powder	100 g	12 weeks	↓ IL-6	[71]
		Diet-induced obese mice	Diet supplemented with cyanidin-3-glucoside, cyanidin-3-rutinoside and pelargonidin-3-glucoside extracted from sweet cherries	20 mg of anthocyanins/kg body weight	16 weeks	↓ IL-6 ↓ Inducible NO synthase ↓TNF-α ↓ NF-κB	[74]
	Human subjects	2 healthy men and 18 healthy women	Daily consumption of sweet cherries	280 g	28 days	↓ CRP ↓ NO	[60]
		2 men and 16 women	Daily consumption of sweet cherries	280 g	28 days	↓ CRP ↓ EGF ↓ Endothelin 1 ↓ EN-RAGE ↓ Ferritin ↓ IL-18 ↓ PAI-1 ↑ IL-1 receptor antagonist ↓ Ferritin	[62]
		10 healthy women	Daily consumption of sweet cherries after overnight fast	280 g	6 days	↓ CRP (after 3 h of sweet cherry consumption) ↓ NO (after 3 h of sweet cherry consumption)	[72]
Cell death and Proliferation	In vitro	A549 cells	Incubation with sweet cherry extract organic fraction	15.62–250 μg/mL	24–72 h	↓ Cell viability	[19]
		HeLa cells	Incubation with sweet cherry extract organic fraction				
			Incubation with sweet cherry crude extract				
		SK-B-NE (2)-C cells	Incubation with sweet cherry crude extract				
		SH-SY5Y cells	Incubation with sweet cherry crude extract				
		SW480 cells	Incubation with undigested cherry extract	121.90 µmol/L (IC50)	24 h	↓ Proliferative activity	[24]
			Incubation with digested cherry extract	61.22 µmol/L		↓ Proliferative activity (more pronounced effect compared to undigested cherry extract)	
		HCT-15 cells	Incubation with digested cherry extract	73.51 µg/mL (IC50)	48 h	↓ Proliferative activity	[18]
		HT29 cells	Incubation with sweet cherry extract	0.5 mg/mL	24-96 h	↓ Proliferative activity	[78]
				0–20 mg dried weight of cherry /mL	96 h		[40]
				0.5 mg/mL	24-96 h	G1/G0 cell cycle arrest	[77]
		MKN45 cells	Incubation with sweet cherry extract	0–20 mg dried weight of cherry /mL	96 h	↓ Cell viability	[40]
		BT-474 cells	Incubation with sweet cherry whole extract	80–320 μg GAE/mL	48 h	↓ Cell growth	[79]
			Incubation with sweet cherry extract enriched in anthocyanins	40–320 μg GAE/mL			
		MDA-MB-231 cells	Incubation with sweet cherry whole extract	80–320 μg GAE/mL	48 h	↓ Cell growth	[79]
			Incubation with sweet cherry extract enriched in anthocyanins	40–320 μg GAE/mL			
			Incubation with sweet cherry extract enriched in proanthocyanins	40–320 μg GAE/mL			
		MDA-MB-453 cells	Incubation with sweet cherry whole extract	80–320 μg GAE/mL	48 h	↓ Cell growth	
				83 μg GAE/mL	8 h	↓ AKT mRNA levels ↓mTOR mRNA levels ↓ p-38-MAPK mRNA levels ↓ Survivin mRNA levels ↓ Sirtuin 1 mRNA levels	
				83 μg GAE/mL	24 h	↑ AKT ↑ Phospho-p-38-MAPK/p38-MAPK protein ratio ↑ Phosphorylated ERK 1/2 ↑ Phosphorylated JNK ↑ mTOR ↓ Phosphorylated mTOR ↓ Phosphorylated mTOR/ mTOR protein ratio ↑ Bax ↑ Bcl-2 ↑ AIF ↑ Cytochrome c ↑ Cleaved caspase-9 ↑ Cleaved caspase-3 ↓ Full PARP ↑ Cleaved PARP ↓ Cleaved PARP/PARP protein ratio	[79,80]
			Incubation with sweet cherry extract enriched in anthocyanins	40–320 μg GAE/mL	48 h	↓ Cell growth	[79]
				70 μg GAE/mL	8 h	↓ AKT mRNA levels ↓mTOR mRNA levels ↓ p-38-MAPK mRNA levels ↓ Survivin mRNA levels ↓ Sirtuin 1 mRNA levels	
				70 μg GAE/mL	24 h	↑ mTOR ↓ Phosphorylated mTOR ↓ Phosphorylated mTOR/ mTOR protein ratio ↓ Full PARP-1 ↑ Cleaved PARP-1 ↓ Cleaved PARP-1/PARP-1 protein ratio	
				19 µg C3G */mL		↑ AKT ↓ Phosphorylated AKT ↑ Phospho-p-38-MAPK/p38-MAPK protein ratio ↑ Phosphorylated ERK 1/2 ↑ Phosphorylated JNK ↑ Cleaved caspase-8 ↑ Bax ↑ Bcl-2 ↑ AIF ↑ Cytochrome c ↑ Cleaved caspase-9 ↑ Cleaved caspase-3 ↓ Full PARP-1 ↑ Cleaved PARP-1	[80]
			Incubation with sweet cherry extract enriched in proanthocyanins	40–320 μg GAE/mL	48 h	↓ Cell growth	[79]
				45 μg GAE/mL	8 h	↓ AKT mRNA levels ↓mTOR mRNA levels ↓ p-38-MAPK mRNA levels ↓ Survivin mRNA levels ↓ Sirtuin 1 mRNA levels	
				45 μg GAE/mL	24 h	↑ mTOR ↓ Phosphorylated mTOR ↓ Phosphorylated mTOR/ mTOR protein ratio ↓ Full PARP ↑ Cleaved PARP ↓ Cleaved PARP/PARP protein ratio	
				22.5 μg PCN */mL		↑ AKT ↑ Phospho-p-38-MAPK/p38-MAPK protein ratio ↑ Phosphorylated ERK 1/2 ↑ Phosphorylated JNK ↑ Bax ↑ Bcl-2 ↑ AIF ↑ Cytochrome c ↑ Cleaved caspase-9 ↑ Cleaved caspase-3 ↓ Full PARP-1 ↑ Cleaved PARP-1	[80]
		PNT1A cells	Incubation with sweet cherry extract	0–200 ug/mL	72 h	↓ Cell viability	[45]
		LNCaP cells		0–200 ug/mL		↓ Cell viability	
				20 µg/mL		↑ Caspase-3 activity ↓ Bcl-2 ↑ Bax/Bcl-2 protein ratio ↑ Caspase-9	
		PC3 cells		0–200 ug/mL		↓ Cell viability	
				20 µg/mL		↓ Caspase-3 activity	
	In vivo	MDA-MB-453 cells xenograft mice model	Oral administration of sweet cherry whole extract	150 mg/kg body weight/day	36 days	↓ Tumor growth ↑ Phosphorylated ERK 1/2 ↓ STAT3 ↓ Ki-67	[81]
			Oral administration of sweet cherry extract enriched in anthocyanins			↓ Tumor growth ↑ Phosphorylated ERK 1/2 ↓ AKT ↓ STAT3 ↓ p38-MAPK ↓ JNK ↓ NF-κB ↓ Ki-67	
			Oral administration of sweet cherry extract enriched in proanthocyanins			↓ Tumor growth ↑ Phosphorylated ERK 1/2 ↓ STAT3 ↓ Ki-67	
Invasion and metastization	In vitro	MDA-MB-453 cells	Incubation with sweet cherry whole extract	83 μg GAE/mL	8 h	↓ Sp1 mRNA levels ↓ Sp4 mRNA levels ↓ VCAM-1 mRNA levels	[79]
				83 μg GAE/mL	24 h	↓ VEGF	[80]
				83 μg GAE/mL	48 h	↓ (?) Cell motility	
			Incubation with sweet cherry extract enriched in anthocyanins	70 μg GAE/mL	8 h	↓ Sp1 mRNA levels ↓ Sp4 mRNA levels ↓ VCAM-1 mRNA levels	[79]
				70 μg GAE/mL	24 h	↓ Sp1	
				19 µg C3G/mL		↓ Migration ↓ PLCγ-1 ↓ VEGF	[80]
				19 µg C3G/mL	48 h	↓ (?) Cell motility	
			Incubation with sweet cherry extract enriched in proanthocyanins	45 μg GAE/mL	8 h	↓ Sp1 mRNA levels ↓ Sp4 mRNA levels ↓ VCAM-1 mRNA levels	[79]
				45 μg GAE/mL	24 h	↓ Sp1	
				22.5 μg PCN/mL		↓ VEGF	[80]
				22.5 μg PCN/mL	48 h	↓ (?) Cell motility	
Metabolic reprogramming	In vitro	PNT1A cells	Incubation with sweet cherry extract	20 µg/mL	72 h	↑ Lactate production ↓ GLUT1 ↓ GLUT3 ↓ PFK-1 ↑ LDH activity ↓ MCT4	[45]
		LNCaP cells				↓ Glucose consumption ↓ Lactate production ↓ GLUT3 ↑ PFK-1 ↓ LDH activity ↓ MCT4	
		PC3 cells				↑ Glucose consumption ↓ PFK-1 ↑ Lactate production ↓ LDH activity	
	In vivo	MDA-MB-453 cells xenograft mice model	Oral administration of sweet cherry extract enriched in anthocyanins	150 mg/kg body weight/day	36 days	Abolished the expression of ACAT1 ↓ lipase E, hormone sensitive type	[81]

* GAE, gallic acid equivalent; C3G, cyanidin 3-glucoside; PCN, proanthocyanins; ↑—stimulatory effect or increased expression, or activity, of specific molecular targets; ↓—suppressor effect or diminished expression, or activity, of specific molecular targets; (?) contradictory information or effect to be confirmed.

## 5. Conclusions

Sweet cherries are one of the most appreciated fruits worldwide because of their pleasant taste and aroma. In addition to their recognized organoleptic properties, sweet cherries also represent a valuable source of nutrients extremely important for several biological functions.

Regardless of the external influencing factors that can determine their specific chemical composition, sweet cherry extracts are very rich in phenolic compounds, with anthocyanins as the main bioactive compounds. The influence of bioactive phytonutrients on biological function highly depends on their bioaccessibility and bioavailability. However, sweet cherry bioactive compounds appear in human blood circulation, intact or as metabolized conjugates. Moreover, consumption of sweet cherries proved, for example, to decrease oxidant circulating species and serum levels of inflammatory markers (Table 2). However, it is well established that digestion decreases the total phenolic compounds, and more realistic models of the digestive process are needed to fully access the bioaccessibility and bioavailability of the sweet cherries’ bioactive compounds and their specific functions.

Over the last few years, the cytoprotective effects of sweet cherries have been extended from the well-known antioxidant and anti-inflammatory actions to the regulation of cell death and proliferation, invasion and migration and the metabolic reprogramming of cancer cells. From the present knowledge, it is quite exciting to conclude the broad action of sweet cherries over several hallmarks of cancer. This has also opened the possibility of strategically using this fruit as a dietary supplement or as a coadjuvant therapy in cancer treatment. However, the existing findings mostly rely on in vitro studies, with animal models and clinical trials being crucial to fully ascertain the anti-cancer effects of sweet cherries.

## Data Availability

No new data were created or analyzed in this study. Data sharing is not applicable to this article.
